# Extra virgin olive oil improves HDL lipid fraction but not HDL-mediated cholesterol efflux capacity: a double-blind, randomised, controlled, cross-over study (OLIVAUS)

**DOI:** 10.1017/S0007114522003634

**Published:** 2023-08-28

**Authors:** Katerina Sarapis, Elena S. George, Wolfgang Marx, Hannah L. Mayr, Jane Willcox, Katie L. Powell, Oladayo S. Folasire, Anna E. Lohning, Luke A. Prendergast, Catherine Itsiopoulos, Colleen J. Thomas, George Moschonis

**Affiliations:** 1 Department of Food, Nutrition and Dietetics, School of Allied Health, Human Services and Sport, La Trobe University, Melbourne, VIC 3086, Australia; 2 Institute for Physical Activity and Nutrition (IPAN), School of Exercise and Nutrition Sciences, Deakin University, Geelong, VIC, Australia; 3 Impact (The Institute for Mental and Physical Health and Clinical Translation), Food & Mood Centre Deakin University, Geelong, VIC, Australia; 4 School of Allied Health, Human Services and Sport, La Trobe University, Melbourne, VIC, Australia; 5 Centre for Functioning and Health Research, Metro South Hospital and Health Service, Brisbane, QLD, Australia; 6 Department of Nutrition and Dietetics, Princess Alexandra Hospital, Brisbane, QLD, Australia; 7 Faculty of Health Sciences & Medicine, Bond University, Robina, QLD, Australia; 8 Centre for Quality and Patient Safety Research, School of Nursing & Midwifery & Institute for Health Transformation, Deakin University, Geelong, VIC, Australia; 9 Department of Mathematics and Statistics, School of Engineering and Mathematical Sciences, La Trobe University, Melbourne, VIC, Australia; 10 School of Health and Biomedical Sciences, RMIT University, Melbourne, VIC, Australia; 11 Department of Microbiology, Anatomy, Physiology and Pharmacology, School of Agriculture, Biomedicine and Environment, La Trobe University, Melbourne, VIC, Australia; 12 Centre for Cardiovascular Biology and Disease Research, School of Agriculture, Biomedicine and Environment, La Trobe University, Melbourne, VIC, Australia; 13 Florey Institute of Neuroscience and Mental Health, Pre-Clinical Critical Care Unit, University of Melbourne, Melbourne, VIC, Australia

**Keywords:** Olive oil, Extra virgin olive oil, Polyphenols, CVD, Cholesterol efflux, HDL-cholesterol, Adults

## Abstract

Olive oil (OO) polyphenols have been shown to improve HDL anti-atherogenic function, thus demonstrating beneficial effects against cardiovascular risk factors. The aim of the present study was to investigate the effect of extra virgin high polyphenol olive oil (HPOO) *v*. low polyphenol olive oil (LPOO) on the capacity of HDL to promote cholesterol efflux in healthy adults. In a double-blind, randomised cross-over trial, fifty participants (aged 38·5 (sd 13·9) years, 66 % females) were supplemented with a daily dose (60 ml) of HPOO (320 mg/kg polyphenols) or LPOO (86 mg/kg polyphenols) for 3 weeks. Following a 2-week washout period, participants crossed over to the alternate treatment. Serum HDL-cholesterol efflux capacity, circulating lipids (i.e. total cholesterol, TAG, HDL, LDL) and anthropometrics were measured at baseline and follow-up. No significant between-group differences were observed. Furthermore, no significant changes in HDL-cholesterol efflux were found within either the LPOO and HPOO treatment arms; mean changes were 0·54 % (95 % CI (0·29, 1·37)) and 0·10 % (95 % CI (0·74, 0·94)), respectively. Serum HDL increased significantly after LPOO and HPOO intake by 0·13 mmol/l (95 % CI (0·04, 0·22)) and 0·10 mmol/l (95 % CI (0·02, 0·19)), respectively. A small but significant increase in LDL of 0·14 mmol/l (95 % CI (0·001, 0·28)) was observed following the HPOO intervention. Our results suggest that additional research is warranted to further understand the effect of OO with different phenolic content on mechanisms of cholesterol efflux via different pathways in multi-ethnic populations with diverse diets.

CVD is the leading cause of mortality globally and accounted for 18·6 million deaths in 2019^(1)^. Dyslipidaemia has a well-established role in the initiation and progression of atheromatous plaque formation and is recognised as an independent risk factor for the development of atherosclerosis-related vascular disease^(2–4)^. Elevated serum levels of LDL-cholesterol and related oxidation products (i.e. oxidised LDL) are involved in the progression of the atherogenic process^(5)^. While serum HDL-cholesterol is widely known to be inversely correlated with CVD, recent data indicate that increased HDL–cholesterol levels do not necessarily imply a reduction in the risk of cardiovascular events and that raised functional capacity of HDL may be responsible for cardiovascular protection^(6)^.

One of the main anti-atherogenic functions of HDL is the regulation of cholesterol homoeostasis through the reverse cholesterol transport pathway, in which excess cholesterol is removed from peripheral cells and transported to the liver for excretion in the bile and faeces^(7,8)^. The efflux of cholesterol from cells to HDL is considered the primary step of reverse cholesterol transport and occurs via two major pathways: ATP-Binding Cassette transporters A1 and G1 (ABCA1 and ABCG1) and the scavenger receptor B1. Specifically, ABCA1 facilitates the efflux of phospholipids and free unesterified cholesterol from cells to lipid-poor apoA-1 through a process that involves the binding of apoA-1 to the ABCA1 transporter, while ABCG1 and scavenger receptor B1 mediate cholesterol efflux from macrophages to HDL^(9–11)^.

Previous evidence indicates that certain diets have beneficial effects on cardiovascular health^(12)^. The traditional Mediterranean diet has been widely reported to be beneficial in the prevention and management of CVD^(13,14)^. Various components of this dietary pattern are cardioprotective, including the high consumption of extra virgin olive oil (EVOO). EVOO has a favourably high content of MUFA as well as important active minor compounds, including polyphenols (i.e. tyrosol, hydroxytyrosol (HT) and oleuropein)^(15,16)^.

Olive oil (OO) polyphenols have been demonstrated to mediate the prevention and management of CVD and associated risk factors (e.g. dyslipidaemia) through various mechanistic pathways^(17)^. In particular, EVOO polyphenols have shown to improve levels of serum HDL-cholesterol^(18)^ as well as the functionality of HDL by reducing HDL oxidative modifications and improving its physiochemical properties^(19–21)^. They also activate ABCA1 expression which is a key protein involved in cholesterol efflux^(19)^.

While numerous *in vitro* and *in vivo* studies have explored the effects of OO on serum lipid levels^(19,22–24)^, there is scarce evidence on the effect of OO polyphenols on HDL-cholesterol efflux capacity. Furthermore, most of the intervention studies are limited to Mediterranean populations that are accustomed to high OO intake^(17)^, thus highlighting the need for additional evidence in multi-ethnic populations with different habitual food cultures. Therefore, the primary aim of the present study was to investigate the effect of 3 weeks daily consumption of either raw extra virgin high polyphenol olive oil (HPOO) (320 mg/kg, phenolic content; 60 ml/d) or low polyphenol olive oil (LPOO) (86 mg/kg, phenolic content; 60 ml/d) on HDL-mediated cholesterol efflux capacity in Australian adults with no previously diagnosed medical conditions. The secondary aim was to compare the effect of the two OO treatments on serum lipids (i.e. total cholesterol, TAG, LDL-cholesterol and HDL-cholesterol).

## Materials and methods

### Study design and procedure

The OLIVAUS study^(25)^ was a double-blind, cross-over, randomised controlled trial that aimed to investigate the effect of extra virgin HPOO compared with a commercially available LPOO on several CVD risk factors in a healthy adult population. Secondary outcomes (i.e. haemodynamic indices, oxidative status and inflammatory markers) have been reported elsewhere as this is not within the scope of this article^(26,27)^. A pilot study was conducted prior to the main study, to test the feasibility of the study protocol and the data collection tools^(28)^. This study was conducted according to the guidelines laid down in the Declaration of Helsinki, and all procedures involving human subjects were approved by the La Trobe University Human Research Ethics Committee (HEC17-067). Written informed consent was obtained from all subjects. The trial protocol has been registered with the Australia New Zealand Clinical Trials Registry (ACTRN12618000706279) and was created in accordance with the SPIRIT statement^(29)^.

Study participants were recruited in Melbourne, Australia through La Trobe University using email advertisements, mailing lists, word of mouth and posters on campus. The inclusion and exclusion criteria used in the OLIVAUS study to identify eligible participants are presented elsewhere^(25)^. Enrolled participants were randomly allocated in a 1:1 ratio, to one of the two treatment arms, that is, extra virgin HPOO or LPOO. Randomisation was performed in blocks of six using a computerised random number generator in excel software. The block randomisation sequence was developed by an independent senior researcher not otherwise involved in the study.

Study participants were instructed to consume a daily dose of 60 ml of either HPOO or LPOO, over two intervention periods of 3 weeks each, added in their usual diet in its raw form. A 3-week study duration was chosen based on previous literature, where most studies were relatively short in duration with most intervention phases lasting on average, 3 weeks^(18)^. Furthermore, the researchers considered potential compliance issues, namely that the study was conducted in a non-Mediterranean population where EVOO is less habitually consumed and accepted. The two kinds of OO had the same nutrient composition (i.e. fat-soluble vitamins and fatty acids) but differed in their phenolic content (320 mg/kg in HPOO *v*. 86 mg/kg in LPOO). Two washout periods of 2 weeks each preceded the first and the second intervention periods of test oils’ administration. During these periods, participants were instructed to avoid consumption of olives and OO. A 2-week washout period was chosen on the basis that this was sufficient to eliminate the carry-over effect of OO polyphenols between interventions, considering the short half-life of OO’s phenolic compounds^(30)^. Furthermore, a daily dose of 60 ml OO was chosen in the current study, since this reflects the habitual amount consumed in Mediterranean populations, where the cardioprotective benefits of virgin OO have previously been reported^(17,18,31)^.

The intervention OO were stored in the same dark, sealed containers, thus ensuring blinding of the participants and researchers. They were supplied to study subjects at the beginning of each intervention period. To ensure further blinding to the type of OO, each container was assigned a different code number that was concealed from study participants and researchers. The code was disclosed only after the completion of the statistical analyses. Participant’s adherence to the intervention was assessed by measuring the volume of unconsumed OO returned at the end of each intervention period. To confirm further adherence, study participants were also asked to record the daily volume of OO consumed over each 3-week intervention period using a log sheet. This information was collected by the researchers at the end of each intervention period. Detailed descriptions and rationale of the study’s protocol and information on the phenolic concentrations and composition of the two intervention OOs are provided elsewhere^(25)^.

### Measurements

#### Socio-demographics, use of medication and dietary supplements

At the first baseline intervention period, study participants’ socio-demographic data and any information related to medication and dietary supplement intake were collected during a scheduled interview by trained researchers using a standardised questionnaire^(25)^.

#### Dietary intake

A 3-d food diary was used to collect dietary intake data during two non-consecutive weekdays and one weekend day at baseline and follow-up of each intervention period. Participants were instructed to record details on the foods and beverages consumed, including the type/brand, quantity in household measures and cooking methods. Emphasis on strategies that incorporate OO into their habitual diet in a raw, uncooked form was provided by the researchers. All dietary intake data were analysed for energy, macro- and micronutrient content using FoodWorks^®^9 software (Xyris Software Pty Ltd). Data related to food groups high in phenolic content (flavonoids, lignans, polyphenols, other polyphenols, stilbenes) that were consumed from study participants during the intervention were also extracted with the use of the nutritional analysis software. A comprehensive database, Phenol-explorer^(32)^, that generates an instant estimate of the polyphenol composition in foods along with their average phenolic concentrations was used to calculate the phenolic content of the above-mentioned food groups and therefore to determine their inclusion in the statistical analyses.

#### Physical activity

Physical activity was assessed during the week preceding the interviews at the first baseline and at the last follow-up meeting using the Active Australia Survey questionnaire, a tool that has been validated in the Australian population^(33)^. This questionnaire is designed to assess participation in a range of leisure time physical activities of light, moderate and vigorous intensity. It consists of eight questions, which assess the number of sessions and total weekly time (hours and/or minutes) spent for each activity type. The amount of time (in minutes per day) that study participants were engaged in physical activity of different intensity was calculated and used for data analysis.

#### Anthropometry

Anthropometric measurements were conducted at baseline and follow-up of each intervention period. Body weight was measured using a digital scale (WM203) to the closest 0·1 kg and with study participants in light clothing and barefoot. Standing height was measured using a wall-mounted stadiometer (SE206) to the nearest 0·1 cm. BMI was calculated using Quetelet’s equation. Study participants were categorised as underweight (BMI < 18·5 kg/m^2^), normal weight (BMI 18·5–24·9 kg/m^2^), overweight (BMI 25·0–29·9 kg/m^2^) or obese (BMI ≥ 30 kg/m^2^)^(34)^. Waist circumference (WC) was measured directly over the skin at the umbilicus level, using a flexible steel tape calibrated in cm with mm graduations (Lufkin W606PM) to the nearest 0·1 cm. Participants were classified as normal (WC < 94 cm in men and <80 cm in women), high CVD risk (WC 94–102 cm in men and 80–88 cm in women) and very high CVD risk (WC > 102 cm in men and 88 cm in women)^(35)^.

#### Biochemical analyses

Biomarkers were measured from early-morning venous blood samples collected from the participants by a trained researcher at baseline and follow-up of each intervention period. Participants attended La Trobe University following a 10-h overnight fast. Collected venous blood was centrifuged (Hettich Rotina 420r) at 2350 rpm for 10 min at 4°C, and the extracted plasma and/or serum was apportioned into aliquots of 500 μl each and stored at −80°C until analysis.

#### HDL-cholesterol efflux capacity

The HDL-cholesterol efflux capacity in serum plasma (collected in serum separating tubes) was measured using the Cholesterol Efflux Fluorometric Assay Kit (BioVision) following the manufacturer’s instructions. Laboratory analysis for cholesterol efflux assay was conducted by AL, KP and OF. Briefly, 5 × 10^4^ J774A.1 (macrophage) cells were seeded into 96-well tissue culture plates and grown in supplemented phenol red-free Gibco Dulbecco’s Modified Eagle Medium for 24 h. Cells were washed with serum-free, phenol red-free Dulbecco’s Modified Eagle Medium and labelled for 1 h in 1:1 ratio labelling reagent to serum-free, phenol red-free Dulbecco’s Modified Eagle Medium. The labelling medium was removed before the cells were incubated in equilibration medium for 18–19 h. Samples were pre-treated with serum treatment reagent containing polyethylene glycol with precipitates apo-B-containing lipoproteins’ to remove interferents (LDL/VLDL) prior to addition to the cells according to the protocol; 2 µl (2 %) of each pre-treated human serum sample, assayed in duplicate, was added and incubated with the cells for 4 h at 37°C. Supernatant was transferred to black walled 96-well plates and fluorescence measured using a FLUOstar Omega spectrophotometer (BMG LabTech) with excitation and emission wavelengths 485 and 520 nm, respectively. Cells were lysed and the lysate was transferred to black-walled 96-well plates and fluorescence measured. Percentage cholesterol efflux was measured using the following equation: *% Cholesterol Efflux = (Relative Fluorescence Units (RFU) of supernatant/RFU of cell lysate + RFU of supernatant) × 100.* The average intra-assay CV was 2·2 %.

#### Total cholesterol, LDL-cholesterol, HDL-cholesterol and TAG

Serum cholesterol concentrations were measured using the Alinity c Cholesterol Assay kit (Abbott GmbH & Co) according to the manufacturer’s instructions. Briefly, cholesterol esters were enzymatically hydrolysed by cholesterol esterase to cholesterol and NEFA (intra-assay CV was 1·0 %). The Alinity c Direct LDL Assay kit (Sekisui Diagnostics P.E.I. Inc) was used for the direct, quantitative determination of LDL-cholesterol in human serum (intra-assay CV was 0·8 %). Serum HDL-cholesterol concentrations were measured using the Ultra HDL Assay kit (Abbott GmbH & Co). This assay is a homogeneous method in which HDL-cholesterol concentrations are measured without the need for off-line pre-treatment or centrifugation steps (intra-assay CV was 1·0 %). Finally, the Alinity c Triglyceride Assay (Abbott GmbH & Co) was used for the quantitation of TAG in human serum. With this method, TAG are enzymatically hydrolysed by lipase to NEFA and glycerol (intra-assay CV was 1·8 %).

### Sample size calculation

The sample size was calculated to detect a 5 % change in the primary outcome, HDL-cholesterol efflux, based on results of previous research^(21)^. A sample size of fifty participants was considered adequate to provide >80 % statistical power to detect significant between-group differences of 5 %, a sd of 11 % in HDL-cholesterol efflux capacity levels and a drop-out rate of 20 %.

### Statistical analysis

All statistical analyses were conducted using the SPSS statistical software for Windows (IBM, version 24.0; IBM). Normality of continuous variables was assessed using the Kolmogorov–Smirnov test. Repeated-measures ANOVA was used to examine: (a) treatment effects (between-group differences, i.e., HPOO *v*. LPOO, at each time point of measurement); (b) time effects (within-group changes in each intervention arm from baseline to follow-up) and (c) treatment × time interaction effects (differences in the changes from baseline to follow-up between the two intervention arms). We performed both per protocol and intention-to-treat analyses. The per protocol analysis included only those participants who had complete data from baseline to follow-up in the first and/or second intervention period. Multiple imputations of missing data were conducted for the intention-to-treat analysis. Because both methods of data analyses provided concordant results in terms of mean values, mean changes and statistical significance, results from the intention-to-treat analysis are presented in this article. Adjustments for sex and age were made in all statistical analyses. Continuous variables are presented either as mean ± sd, as estimated marginal means and se or as mean change and 95 % CI of change. In addition, categorical variables are presented as frequency (*n*) and percentage. Statistical significance is set at *P* < 0·05 and all reported *P* values are two-tailed.

## Results

**Fig. 1.**
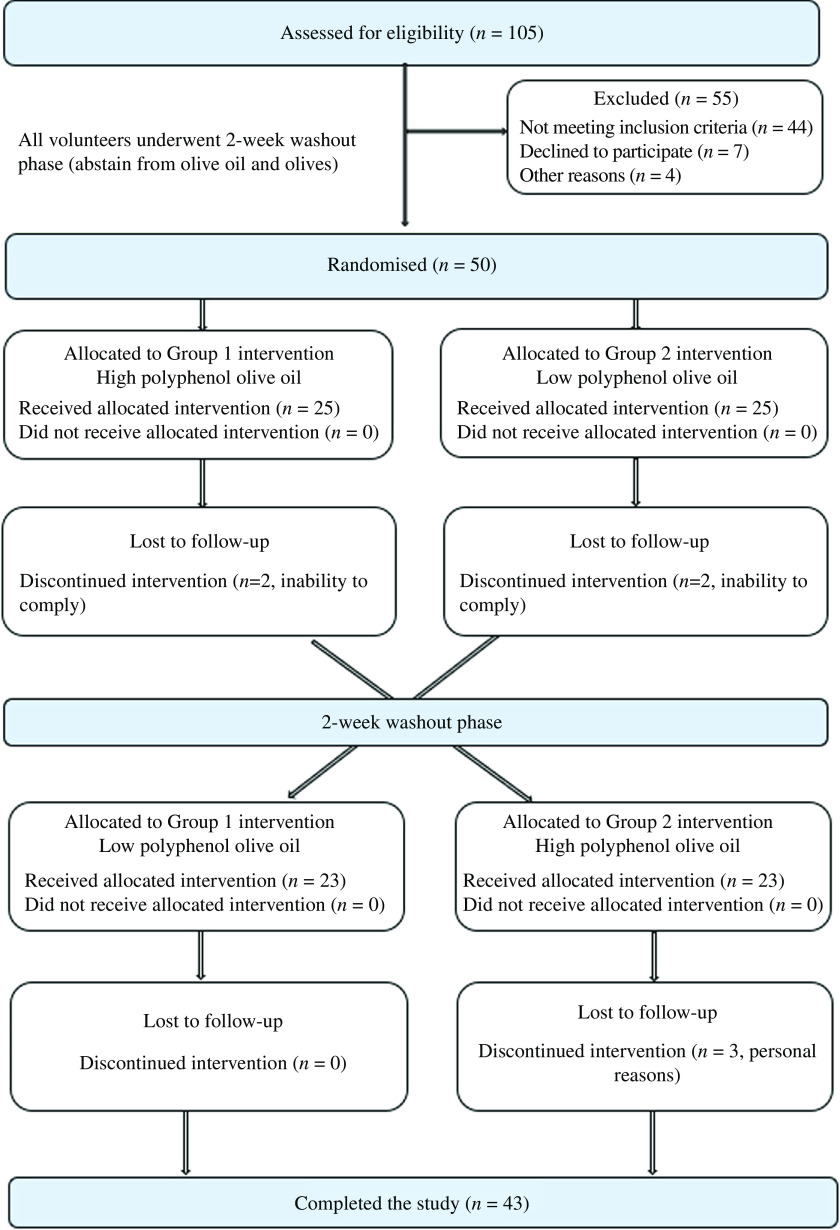
OLIVAUS study participant flow diagram.

Fifty volunteers (*n* 33 females and *n* 17 males) were enrolled in the study from July 2018 to October 2019 and were randomly allocated in a 1:1 ratio, to one of the two treatment arms, that is, HPOO or LPOO. Following a 2-week washout period, participants crossed over to the alternate treatment. Four participants (*n* 2, HPOO and *n* 2, LPOO) discontinued the study post first intervention phase due to inability to comply (i.e. found challenging to consume 60 ml/d OO in its raw, uncooked form) and three participants (*n* 3, HPOO) withdrew post second intervention phase for personal reasons. In total, forty-three participants (86 %) completed the study (Fig. 1). Furthermore, participant compliance to treatment was overall high and did not differ significantly between the two treatment arms as shown in online Supplementary Table 1.

### Baseline characteristics of study participants

Baseline characteristics of the total sample (*n* 50) and by treatment arm in terms of socio-demographics, anthropometric and biochemical indices are presented in Table 1. The mean age of participants was 39 (sd 14) years (age range, 20–70 years), and the majority were born in Australia (70 %). The mean BMI and WC of participants were 24·7 (sd 3·5) kg/m^2^ and 86·9 (sd 11·2) cm, respectively. In addition, 48 % of the study population was classified as overweight or obese, while 16 % of study participants were considered at high CVD risk and 34 % very high CVD risk according to their WC measurements. Mean serum HDL-cholesterol efflux capacity concentration was 53·1 (sd 4·8) % for the total cohort. Mean circulating TAG, total cholesterol, HDL- and LDL-cholesterol were 1·0 (sd 0·5) mmol/l, 5·0 (sd 0·5) mmol/l, 1·5 (sd 0·3) mmol/l and 3·0 (sd 0·9) mmol/l, respectively.

**Table 1.** Baseline descriptive characteristics of study participants (Mean values and standard deviations)

	Total sample (*n* 50)		Low polyphenol OO (*n* 25)		High polyphenol OO (*n* 25)		*P* *
	Mean	SD	Mean	SD	Mean	SD	
Socio-demographics							
Age (years)	38·5	13·9	38·1	14·8	39·0	13·2	0·818
Education (years)	17·3	3·5	17·3	4·2	17·2	2·8	0·968
Sex							
	%		%		%		
Females	66		64		68		0·765
Males	34		36		32		
Region of birth							
	%		%		%		
Australia, NZ, Pacific	70		68		72		0·321
Islanders							
Europe	10		4		16		
South America	8		12		4		
Middle East and Asia	12		16		8		
Anthropometrics							
Height (cm)	168·9	9·6	170·6	10·4	167·2	8·6	0·22
Weight (kg)	70·7	12·8	72·7	13·7	68·5	11·8	0·249
BMI (kg/m^2^)	24·7	3·5	24·9	3·7	24·4	3·2	0·617
Waist circumference (cm)	86·9	11·2	88·2	11·9	85·6	10·6	0·434
Weight status categories†							
	%		%		%		
Underweight	2		0		4		0·252
Normal weight	50		56		44		
Overweight	44		36		52		
Obese	4		8		0		
Waist circumference categories‡							
	%		%		%		
Normal	50		44		56		0·632
High risk	16		20		12		
Very high risk	34		36		32		
Biochemical indices							
HDL-cholesterol efflux (%)	53·1	4·8	52·7	4·6	53·3	5·0	0·663
TAG (mmol/l)	1·0	0·5	1·1	0·7	0·9	0·3	0·334
TC (mmol/l)	5·0	1·0	4·9	1·1	5·2	0·9	0·37
HDL-cholesterol (mmol/l)	1·5	0·3	1·5	0·3	1·6	0·3	0·15
LDL-cholesterol (mmol/l)	3·0	0·9	3·0	0·9	3·1	0·8	0·582

OO, olive oil; NZ, New Zealand; TC, total cholesterol.

* *P*-values for testing between-group differences in continuous variables were derived from the independent samples *t*-test. *P*-values for examining associations between categorical variables were derived from the *χ* ^2^ test.

† Weight status categories: underweight, BMI < 18·5 kg/m^2^; normal weight, 18·5 ≤ BMI < 25 kg/m^2^; overweight, 25 ≤ BMI < 30 kg/m^2^; obese, BMI ≥ 30 kg/m^2(34)^.

‡ Waist circumference categories: normal, WC < 80 cm in women and <94 cm in men; high risk, 80–88 cm in women and 94–102 cm in men; very high risk: WC > 88 cm in women and > 102 cm in men^(35)^.

### Effect of low polyphenol olive oil and high polyphenol olive oil on dietary intake and physical activity

As reported previously, no significant between-group differences were observed in dietary energy, macro- and micronutrient intake from baseline to follow-up^(26,27)^. A significant increase in dietary energy intake was observed within both the LPOO (by 1806·1 kJ/d, 95 % CI 1075·4, 2536·8) and HPOO (by 1766·6 kJ/d, 95 % CI 1035·9, 2497·3) treatment arms. Similarly, consumption of LPOO and HPOO led to a significant increase in total fat (by 49·3 g/d, 95 % CI 41·1, 57·4 and 46·0 g/d, 95 % CI 37·8, 54·1, respectively), SFA (by 7·4 g/d, 95 % CI 4·0, 10·8 and 6·5 g/d, 95 % CI 3·1, 9·9, respectively), MUFA (by 36·8 g/d, 95 % CI 33·2, 40·3 and 35·1 g/d, 95 % CI 31·6, 38·6, respectively) and PUFA intake (by 3·1 g/d, 95 % CI 1·0, 5·1 and 3·0 g/d, 95 % CI 1·0, 5·1, respectively) from baseline to follow-up.

Data related to food groups high in phenolic content (i.e. whole grain cereals, fruits, vegetables, legumes, nuts/seeds, soya products, oils, fruit juices, alcoholic drinks and coffee) that participants consumed during the intervention (online Supplementary Table 2) were also analysed. Results demonstrated no significant between-group differences or within-group changes in the intake of the above-mentioned food items/beverages from baseline to follow-up. However, a significant increase in oil intake was observed within both treatment arms as expected due to the increased intake of the intervention oils (online Supplementary Table 3). Regarding physical activity, no within-group changes or between-group differences were observed in daily energy expenditure in leisure time physical activity over the intervention period (data not shown).

### Effect of low polyphenol olive oil and high polyphenol olive oil on anthropometric data

No significant between-group differences were observed in any of the examined anthropometric outcomes (i.e. weight, BMI, WC) (online Supplementary Table 4). A small but significant increase in body weight by 0·4 kg (95 % CI 0·2, 0·7) was observed only within the LPOO treatment arm. No within-group changes were observed in any of the other outcomes (i.e. BMI and WC).

### Effect of low polyphenol olive oil and high polyphenol olive oil on HDL-cholesterol efflux capacity and serum lipids

**Fig. 2.**
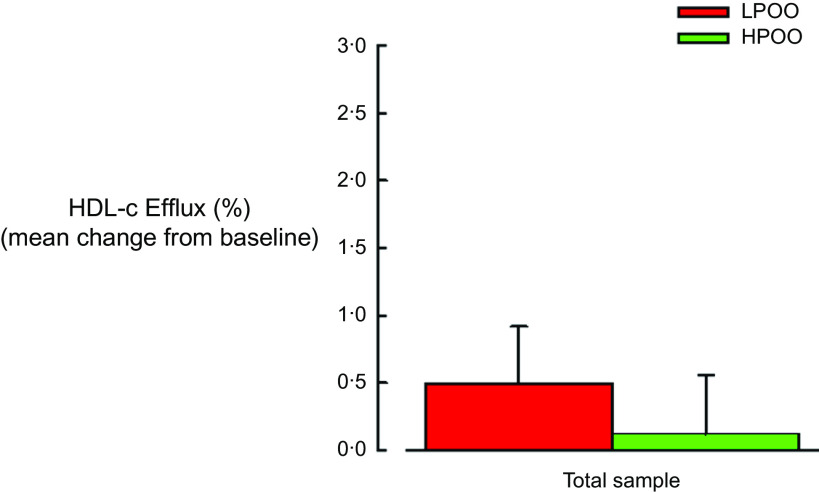
Effect of 3-week daily consumption of extra virgin high polyphenol olive oil (HPOO; 320 mg/kg polyphenols; *n* 43) and low polyphenol olive oil (LPOO; 86 mg/kg polyphenols; *n* 44) on HDL-cholesterol efflux. No within-group changes or between-group differences were observed in HDL-cholesterol efflux. Data are expressed as mean changes ± standard errors from baseline to follow-up.

The effect of the two OO interventions on HDL-cholesterol efflux capacity is illustrated in Fig. 2. No significant between-group differences were observed regarding the changes in HDL-cholesterol efflux capacity from baseline to follow-up. There were no significant changes in HDL-cholesterol efflux capacity observed within either the LPOO and HPOO groups; mean changes were 0·54 % (95 % CI 0·29, 1·37) and 0·10 % (95 % CI 0·74, 0·94), respectively, for the total sample. No between-group differences were observed in circulating TAG, total cholesterol, HDL-cholesterol and LDL-cholesterol for the total sample from baseline to follow-up (Table 2). However, compared with baseline, serum HDL-cholesterol significantly increased after LPOO and HPOO intake by 0·13 mmol/l (95 % CI 0·04, 0·22) and 0·10 mmol/l (95 % CI 0·02, 0·19), respectively. There was a small but significant increase in LDL-cholesterol by 0·14 mmol/l (95 % CI 0·001, 0·28) following the HPOO intervention; however, no significant differences were observed between the two treatment arms. No significant within-group changes or between-group differences were observed in TAG and total cholesterol.

**Table 2.** Effect of low polyphenol OO *v*. high polyphenol OO on mean changes in serum lipids (Mean values with their standard error of the means; 95 % confidence intervals)

Biomarker	Baseline		Follow-up		Change (time-effect)	95 % CI	Treatment × time interaction
	Mean	sem	Mean	sem			*P*
TAG (mmol/l)							
Low polyphenol OO (*n* 50)	0·97	0·07	0·89	0·09	−0·08	−0·21, 0·05	0·425
High polyphenol OO (*n* 50)	0·92	0·07	0·91	0·09	−0·01	−0·14, 0·12	
Treatment × effect *P*-value	0·613		0·860				
Total cholesterol (mmol/l)							
Low polyphenol OO (*n* 50)	5·01	0·13	5·08	0·13	0·08	−0·11, 0·27	0·856
High polyphenol OO (*n* 50)	5·09	0·13	5·14	0·13	0·05	−0·14, 0·24	
Treatment × effect *P*-value	0·646		0·749				
HDL-cholesterol (mmol/l)							
Low polyphenol OO (*n* 50)	1·57	0·05	1·70	0·05	0·13**	0·04, 0·22	0·650
High polyphenol OO (*n* 50)	1·56	0·05	1·66	0·05	0·10*	0·02, 0·19	
Treatment × effect *P*-value	0·927		0·627				
LDL-cholesterol (mmol/l)							
Low polyphenol OO (*n* 50)	3·01	0·11	3·04	0·11	0·03	−0·11, 0·16	0·256
High polyphenol OO (*n* 50)	3·05	0·11	3·19	0·11	0·14*	0·001, 0·28	
Treatment × effect *P*-value	0·795		0·330				

OO, olive oil.

All statistical analyses were adjusted for sex and age.

* Indicates statistical significance (*P* < 0.05).

## Discussion

The present double-blind, cross-over, randomised controlled trial investigated the effect of 3-week daily consumption of either raw extra virgin HPOO (320 mg/kg, phenolic content; 60 ml, daily dose) or LPOO (86 mg/kg, phenolic content; 60 ml daily dose) on HDL-cholesterol efflux capacity and serum lipids in Australian adults with no previously diagnosed medical condition. Our results showed no significant differences in any of the examined biomarkers between the two treatment arms. A non-significant mean increase in HDL-cholesterol efflux capacity was observed within both the LPOO and HPOO groups for the total sample, while serum HDL-cholesterol increased significantly within both treatment arms. A small but significant increase in circulating LDL-cholesterol was also observed in the HPOO arm.

Although an increase in HDL-cholesterol efflux capacity was observed within both arms in our study (+0·54 %, LPOO; +0·10 %, HPOO), this did not reach statistical significance which is inconsistent with previous studies. For example, Hernaez *et al.* ^(21)^ demonstrated that the daily consumption of 25 ml raw HPOO (366 mg/kg, phenolic content) or LPOO (2·7 mg/kg, phenolic content) for 3 weeks significantly increased HDL-cholesterol efflux capacity by 3·05 % only in the HPOO treatment arm compared with the LPOO arm (-2·34 %) in healthy men. A significant increase in the capacity of HDL to promote cholesterol efflux by 14·81 % was also reported for healthy adults, after 12 weeks of EVOO intake (793 mg/kg, phenolic content; 25 ml daily dose) in a non-controlled single-arm study^(9)^. Supporting these data, another study showed that the consumption of a functional virgin OO enriched with OO and thyme phenolic compounds (500 mg/kg, total phenolic content; 25 ml daily dose) for 3 weeks, compared with a virgin OO (80 mg/kg, phenolic content), enhanced the ability of HDL to induce cholesterol efflux from macrophages via the increased expression of cholesterol efflux-related genes in hypercholesterolaemic adults^(36)^. The differences in phenolic content of the intervention oils used in the aforementioned clinical trials compared with our study could potentially explain the different reported effects.

A beneficial effect of HPOO intake on HDL-cholesterol efflux capacity was also supported by a recent systematic review and meta-analysis, which examined the effect of HPOO *v*. LPOO (150–800 mg/kg *v*. 0–132 mg/kg, phenolic content, respectively; 25–75 ml, daily dose)^(18)^. However, most of the studies included in this meta-analysis were conducted in Mediterranean populations, who are accustomed to high OO consumption. In addition, previous research has primarily assessed the effect of EVOO as part of the MedDiet^(37)^, further suggesting that many of the OO attributed benefits might be also related to other elements of this diet (i.e. fruits, vegetables) that have a proven cardioprotective effect and/or the synergistic effects between EVOO and other plant-derived polyphenols. These observations highlight that additional studies with diverse ethnicities and food cultures are required to confirm the reported beneficial effects of HPOO and determine if there are also genetic differences that may predispose individuals to the cardiovascular benefits associated with polyphenol intake.

The molecular mechanisms by which phenolic compounds promote cholesterol efflux still require further elucidation. These mechanisms involve the passive diffusion process as well as pathways that are mediated by the transmembrane transporters ABCA1, ABCG1 and the scavenger receptor B1. Specifically, ABCA1 facilitates cholesterol efflux from cells to lipid-poor apolipoproteins while ABCG1 and the scavenger receptor B1 receptor are responsible for the efflux of cholesterol from macrophages to HDL^(38,39)^. EVOO phenolic compounds, especially HT, have been shown to stimulate ABCA1 protein expression, enhancing cholesterol efflux to lipid poor apoA1-containing lipoprotein particles via direct binding to ABCA1^(40)^. Sola *et al.* ^(41)^ reported an increase in apoA-1 concentrations after virgin OO consumption in high-CVD risk individuals; Violante *et al.* ^(42)^ also demonstrated that consumption of EVOO for 3 months increased serum apoA-1 in hypercholesterolaemic subjects. Given that participants in the present study were healthy and without any previously diagnosed medical condition, this may be one explanation for the non-significant changes in HDL-cholesterol efflux following both LPOO and HPOO intake.

The effect of the two kinds of OO on serum lipids was also examined in the current study. Results demonstrated that both extra virgin HPOO and LPOO consumption significantly increased circulating HDL-cholesterol after 3 weeks. There is evidence supporting the effect of OO polyphenols on serum HDL-cholesterol; however, the data are once again inconsistent. In 2015, a meta-analysis reported no effect of EVOO intake (150 mg/kg, phenolic content) on circulating HDL-cholesterol in both healthy and adults with CVD^(17)^, while another systematic review demonstrated that EVOO intake (2·28–75 g daily dose) increased the levels of serum HDL-cholesterol in dyslipidaemic subjects^(2)^. A dose-dependent increase of circulating HDL-cholesterol was also reported in the EUROLIVE study, a European multicentre study, after consumption of three types of OO differing in their phenolic content (2·7 mg/kg, phenolic content; 164 mg/kg, phenolic content and 366 mg/kg, phenolic content, respectively)^(22)^. Another study however demonstrated that increased concentration of OO polyphenols in the lipoprotein fraction may increase HDL particle size, stability and antioxidant status but not circulating HDL-cholesterol levels.

It has been reported that the daily consumption of OO rich in polyphenols reduces LDL-cholesterol and improves lipoprotein-associated atherogenic ratios^(43)^. In contrast, another study reported that 1-year intervention with a MedDiet enriched with EVOO improved various LDL atherogenic related characteristics but did not improve the plasma LDL-cholesterol concentrations in a sub-sample of adults at high CVD risk^(44)^. Our results are consistent with this positive association between OO intake and LDL-cholesterol levels. However, as recently also reported by the current study^(27)^, the levels of ox-LDL were found to be reduced in the HPOO treatment arm, thus highlighting that the increase in LDL-cholesterol does not necessarily imply increased atherogenic risk.

With regard to the dietary intake changes and any potential confounding effect caused by differences in dietary intake, the OLIVAUS study showed that both treatment oils equally increased the intake of energy and macronutrients (i.e. fatty acids), while no significant within-group changes or between-group differences were observed in the intake of foods high in polyphenols (online Supplementary Tables 2 and 3). It is noteworthy that both the LPOO and HPOO tested in the current study had the same nutritional composition in terms of fat-soluble vitamins and fatty acids, so the observed improvements in the examined biomarkers should be exclusively associated with the intervention oils’ phenolic content. However, the concentration of HT, which is the most biologically active phenolic compound found in virgin OO^(45)^, was higher in the LPOO (5·3 mg/kg, HT) compared with HPOO (3·3 mg/kg, HT) in the current study, possibly explaining the more pronounced increase in HDL-cholesterol efflux capacity in the LPOO treatment arm. This finding also suggests that further research is warranted to explore the effects of different phenols on CVD risk markers.

The findings of the present study should be interpreted in light of its strengths and limitations. A strength of this study was its randomised, cross-over double-blind, controlled design, which confers strong inter-individual variability. Another strength was that study participants retained their habitual diet, hence allowing us to directly assess the effects of OO intake. Furthermore, our findings indicate that a moderate concentration of OO polyphenols (i.e. 86 mg/kg) can still induce positive effects on CVD risk markers. These results, if verified in future studies, might have a direct impact on the OO industry and human nutrition. One of the study’s limitations is that although participant compliance to the intervention was overall high, measurements of compliance relied on self-reporting methods and were therefore subjective. Another limitation is the fact that there was only a 4-fold difference in the phenolic content between the HPOO and LPOO (320 mg/kg *v*. 86 mg/kg, respectively), compared with other studies that showed significant effects on HDL efflux but had 6 to 14-fold difference in the respective phenolic content. Lastly, despite the inclusion of a washout period before the initiation of the intervention and between the intervention periods, there is no guarantee that any potential carry-over effect on the examined biomarkers was completely avoided. However, pairwise comparisons that examined potential carry-over effects were insignificant for all biomarkers.

### Conclusions

The OLIVAUS study examined the effect of OO polyphenols on HDL-cholesterol efflux capacity and serum lipids in healthy Australian adults. No significant differences between the two OO treatment arms were observed in any of the examined outcomes. A non-significant mean increase in serum HDL-cholesterol efflux capacity was observed within both the LPOO and extra virgin HPOO treatment arms after consumption of the two kinds of OO for 3 weeks each. Furthermore, a significant increase in circulating HDL-cholesterol was observed within both treatment arms. However, the non-significant findings of our study indicate that additional studies of longer duration are warranted to further understand the effect of OO polyphenols of different content and/or profile on mechanisms of cholesterol efflux via different pathways, especially in a multi-ethnic population with different food cultures.

## Supplementary material

For supplementary material accompanying this paper visit https://doi.org/10.1017/S0007114522003634. click here to view supplementary material
