# Supplementary material for: Extra virgin olive oil improves HDL lipid fraction but not HDL-mediated cholesterol efflux capacity: a double-blind, randomised, controlled, cross-over study (OLIVAUS)
Source: Br J Nutr. 2022 Nov 15;130(4):641–50. doi: 10.1017/S0007114522003634 (PMC10357319; doi:10.1017/S0007114522003634)
Supplement: Supplementary file 1 [file S0007114522003634sup001.docx]

**Supplementary Table 1:** Summary of olive oil (OO) volume returned by study participants following the two diet interventions.

|  | **Low polyphenol OO** | **High Polyphenol OO** |  |
| --- | --- | --- | --- |
|  | **Mean (SD)** | **Mean (SD)** | **P-value*** |
| **1^st^ intervention** |  |  |  |
| Actual remaining OO (ml) | 106.4 (152.2) | 100.4 (123.6) | 0.888 |
| Compliance based on actual remaining (%) | 92 | 92 |  |
| **2^nd^ Intervention** |  |  |  |
| Actual remaining OO (ml) | 105.9 (133.2) | 123.1 (164.7) | 0.708 |
| Compliance based on actual remaining (%) | 92 | 90 |  |

*P-values derived from the non-parametric Mann-Whitney test. SD, standard deviation; OO, olive oil. Low or high polyphenol OO (1260 ml, total volume) was supplied at the commencement of each 3-week intervention period.

**Supplementary Table 2:** Food groups high in phenolic content (Phenol Explorer)

| **Food Groups** | **Phenolic Content (mg/100 mL)*** |
| --- | --- |
| **Grains** |  |
| Whole grains | 167.24 |
| Refined grains | 49.18 |
| **Vegetables** |  |
| Red/orange vegetables | 260.71 |
| Green vegetables | 129.58 |
| Other vegetables (white, yellow, pink, black, purple) | 50.19 |
| **Fruits** |  |
| Citrus/berries | 16.51 |
| **Legumes** |  |
| Lentils, chickpeas, beans | 114.92 |
| **Nuts** |  |
| Almonds, peanuts, walnuts, macadamia, pines, pistachio | 44.83 |
| **Oils** |  |
| Cereal, vegetable, nut, seed | 21.10 |
| **Beverages** |  |
| Coffee | 14.85 |
| Fruit Juice | 6.98 |
| Tea Infusions | 3.14 |
| Alcoholic drinks (wine, beer, spirits) | 1.01 |

* Data related to food groups high in phenolic content (flavonoids, lignans, polyphenols, other polyphenols, stilbenes) that participants consumed during the intervention, were extracted from FoodWorks nutritional analysis software. Phenol-explorer ^(32)^ was used to calculate the phenolic content of the above-mentioned food groups and therefore to determine their inclusion

in the statistical analyses.

**Supplementary Table 3:** Effect of Low Polyphenol Olive Oil (OO) vs. High Polyphenol Olive Oil (OO) on dietary

intake (Food groups)

| **Dietary intake** | **Baseline** | **Follow-up** | **Change (Time-effect)** | **Treatment*time interaction** |
| --- | --- | --- | --- | --- |
|  | **Mean (SEM)** | **Mean (SEM)** | **(95% CI)** | **(p-value)** |
| **Grains (Serves)** |  |  |  |  |
| Low Polyphenol OO (n=50) | 7.2 (0.5) | 7.2 (0.5) | 0.05 (-0.9 to 1.0) | 0.811† |
| High Polyphenol OO (n=50) | 7.5 (0.5) | 7.4 (0.5) | -0.1 (-1.1 to 0.9) |  |
| Treatment*effect p-value | 0.581 | 0.796 |  |  |
| **Refined Grains (Serves)** |  |  |  |  |
| Low Polyphenol OO (n=50) | 4.1 (0.4) | 4.1 (0.4) | 0.0 (-0.9 to 0.8) | 0.931† |
| High Polyphenol OO (n=50) | 4.4 (0.4) | 4.3 (0.4) | -0.1 (-0.9 to 0.7) |  |
| Treatment*effect p-value | 0.6 | 0.679 |  |  |
| **Whole Grains (Serves)** |  |  |  |  |
| Low Polyphenol OO (n=50) | 3.1 (0.3) | 3.2 (0.4) | 0.1 (-0.4 to 0.8) | 0.063‡ |
| High Polyphenol OO (n=50) | 3.1 (0.3) | 3.1 (0.4) | 0.0 (-0.6 to 0.6) |  |
| Treatment*effect p-value | 0.392 | 0.462 |  |  |
| **Fruits (Serves)** |  |  |  |  |
| Low Polyphenol OO (n=50) | 1.3 (1.1) | 1.4 (1.1) | 0.1 (-0.2 to 0.3) | 0.431‡ |
| High Polyphenol OO (n=50) | 1.6 (1.1) | 1.5 (1.1) | -0.1 (-0.3 to 0.2) |  |
| Treatment*effect p-value | 0.219 | 0.642 |  |  |
| **Citrus/melons/berries (Serves)** | |  |  |  |
| Low Polyphenol OO (n=50) | 0.3 (0.1) | 0.3 (0.0) | 0.0 (-0.1 to 0.1) | 0.855‡ |
| High Polyphenol OO (n=50) | 0.3 (0.1) | 0.3 (0.0) | 0.0 (-0.1 to 0.1) |  |
| Treatment*effect p-value | 0.24 | 0.321 |  |  |
| **Fruit juice (Serves)** |  |  |  |  |
| Low Polyphenol OO (n=50) | 0.1 (0.0) | 0.1 (0.0) | 0.0 (-0.1 to 0.1) | 0.967‡ |
| High Polyphenol OO (n=50) | 0.1 (0.0) | 0.2 (0.0) | 0.1 (-0.1 to 0.2) |  |
| Treatment*effect p-value | 0.372 | 0.399 |  |  |
| **Vegetables (Serves)** |  |  |  |  |
| Low Polyphenol OO (n=50) | 4.1 (0.3) | 4.2 (0.5) | 0.1 (-1.0 to 1.2) | 0.635‡ |
| High Polyphenol OO (n=50) | 4.1 (0.5) | 4.8 (0.5) | 0.7 (-0.4 to 1.8) |  |
| Treatment*effect p-value | 0.928 | 0.61 |  |  |
| **Dark Green Vegetables (Serves)** | |  |  |  |
| Low Polyphenol OO (n=50) | 0.5 (0.1) | 0.7 (0.2) | 0.2 (-0.2 to 0.6) | 0.394‡ |
| High Polyphenol OO (n=50) | 0.5 (0.1) | 0.9 (0.2) | 0.4 (0.0 to 0.8) |  |
| Treatment*effect p-value | 0.485 | 0.686 |  |  |
| **Red/orange vegetables (Serves)** | |  |  |  |
| Low Polyphenol OO (n=50) | 1.3 (0.2) | 1.2 (0.2) | -0.1 (-0.6 to 0.4) | 0.103‡ |
| High Polyphenol OO (n=50) | 1.2 (0.2) | 1.6 (0.2) | 0.4 (-0.1 to 0.8) |  |
| Treatment*effect p-value | 0.697 | 0.078 |  |  |
| **Legumes (Serves)** |  |  |  |  |
| Low Polyphenol OO (n=50) | 0.5 (0.2) | 0.5 (0.2) | 0.0 (-0.3 to 0.4) | 0.544‡ |
| High Polyphenol OO (n=50) | 0.5 (0.1) | 0.5 (0.1) | -0.1 (-0.4 to 0.3) |  |
| Treatment*effect p-value | 0.719 | 0.739 |  |  |
| **Nuts/Seeds (Serves)** |  |  |  |  |
| Low Polyphenol OO (n=50) | 0.6 (0.1) | 0.6 (0.1) | 0.0 (-0.2 to 0.3) | 0.333‡ |
| High Polyphenol OO (n=50) | 0.7 (0.1) | 0.7 (0.1) | 0.0 (-0.3 to 0.2) |  |
| Treatment*effect p-value | 0.321 | 0.992 |  |  |
| **Soy Products (Serves)** |  |  |  |  |
| Low Polyphenol OO (n=50) | 0.1 (0.1) | 0.1 (0.0) | 0.0 (-0.1 to 0.1) | 0.444‡ |
| High Polyphenol OO (n=50) | 0.1 (0.1) | 0.1 (0.0) | 0.0 (-0.1 to 0.1) |  |
| Treatment*effect p-value | 0.259 | 0.797 |  |  |
| **Oil (tsp)** |  |  |  |  |
| Low Polyphenol OO (n=50) | 9.1 (0.6) | 19.7 (0.7) | **10.6 (9.3 to 12.0)** | 0.589† |
| High Polyphenol OO (n=50) | 10.0 (0.7) | 20.1 (0.7) | **10.1 (8.8 to 11.4)** |  |
| Treatment*effect p-value | 0.337 | 0.703 |  |  |
| **Alcoholic drinks (stand. drinks)** | |  |  |  |
| Low Polyphenol OO (n=50) | 1.1 (0.4) | 0.5 (0.2) | -0.5 (-1.4 to 0.4) | 0.774‡ |
| High Polyphenol OO (n=50) | 0.6 (0.4) | 0.8 (0.2) | 0.2 (-0.7 to 1.0) |  |
| Treatment*effect p-value | 0.928 | 0.84 |  |  |
| **Caffeine intake (mg/day)** |  |  |  |  |
| Low Polyphenol OO (n=50) | 199.4 (54.1) | 182.0 (35.6) | -17.4 (-122.3 to 87.6) | 0.612† |
| High Polyphenol OO (n=50) | 242.8 (56.0) | 186.7 (36.8) | -56.1 (-164.6 to 52.5) |  |
| Treatment*effect p-value | 0.578 | 0.926 |  |  |

All statistical analyses were adjusted for gender and age. Results in bold indicate P <0.05, therefore statistically significant. The normality of the distribution of each food intake variable was examined with the Kolmogorov-Smirnov test.

^†^ The statistical test that was used to estimate the treatment effect and treatment*time effect P values on normally distributed data was repeated measures ANOVA.

^‡^ The statistical test that was used to estimate the treatment effect and treatment*time effect P values on non-normally distributed data was the non-parametric Friedman test for repeated measures.

**Supplementary Table 4:** Effect of low polyphenol OO vs. high polyphenol OO on mean changes in anthropometric indices.

|  | **Baseline**  **Mean (SEM)** | **Follow-up**  **Mean (SEM)** | **Change (Time-effect)**  **(95% CI)** | **Treatment*time interaction**  **(p-value)** |
| --- | --- | --- | --- | --- |
| **Weight (kg)** |  |  |  |  |
| Low Polyphenol OO (n=50) | 70.8 (1.5) | 71.2 (1.5) | **0.4 (0.2 to 0.7)** | **0.163** |
| High Polyphenol OO (n=50) | 70.7 (1.5) | 70.9 (1.5) | 0.2 (-0.1 to 0.4) |  |
| Treatment*effect p-value | 0.993 | 0.902 |  |  |
| **Height (cm)** |  |  |  |  |
| Low Polyphenol OO (n=50) | 168.9 (0.9) | 169.0 (0.9) | 0.1 (-0.2 to 0.4) | 0.890 |
| High Polyphenol OO (n=50) | 168.9 (0.9) | 169.0 (0.9) | 0.1 (-0.1 to 0.4) |  |
| Treatment*effect p-value | 0.974 | 0.992 |  |  |
| **BMI (kg/m^2^)** |  |  |  |  |
| Low Polyphenol OO (n=50) | 24.7 (0.4) | 24.8 (0.4) | 0.1 (-0.01 to 0.2) | 0.305 |
| High Polyphenol OO (n=50) | 24.7 (0.4) | 24.7 (0.4) | 0.02 (-0.1 to 0.1) |  |
| Treatment*effect p-value | 0.993 | 0.897 |  |  |
| **Waist circumference (cm)** |  |  |  |  |
| Low Polyphenol OO (n=50) | 87.1 (1.3) | 87.4 (1.2) | 0.3 (-0.1 to 0.7) | 0.501 |
| High Polyphenol OO (n=50) | 87.1 (1.3) | 87.3 (1.2) | 0.1 (-0.2 to 0.5) |  |
| Treatment*effect p-value | 1.000 | 0.919 |  |  |

All statistical analyses were adjusted for gender and age. Results in bold indicate statistical significance (p < 0.05). OO, olive oil; SEM, standard error of the mean; CI, confidence interval.
